# The EU Response to the Presence of Nitrosamine Impurities in Medicines

**DOI:** 10.3389/fmed.2021.782536

**Published:** 2021-11-19

**Authors:** Robin Ruepp, Roland Frötschl, Robert Bream, Maria Filancia, Thomas Girard, Andrei Spinei, Martina Weise, Rhys Whomsley

**Affiliations:** ^1^European Medicines Agency, Human Medicines Division, Amsterdam, Netherlands; ^2^Licensing Division 2, Federal Institute for Drugs and Medical Devices (Bundesinstitiut für Arzneimittel und Medizinprodukte), Bonn, Germany

**Keywords:** nitrosamines, human medicines, sartans, ranitidine, metformin, nitrite (NaNO_2_), carcinogenicity, linear extrapolation

## Abstract

The unexpected detection of nitrosamine impurities in human medicines has recently seen global regulators act to understand the risks of these contaminations to patients and to limit their presence. Over 300 nitrosamines are known, many of which are highly potent mutagenic carcinogens. Regulators first became aware of the presence of nitrosamines in EU medicines in 2018, with reports of detection of *N*-nitroso-dimethylamine (NDMA) in valsartan from one manufacturer. A subsequent EU review of all valsartan medicines was triggered by the European Medicines Agency (EMA) and was later extended to other angiotensin receptor blockers/sartans. A separate review was also started for ranitidine medicines. This was followed by an EU-wide examination of the risk of presence of nitrosamines in all human medicines. This article reflects on the investigation of the EU regulatory network into the presence of nitrosamines and the scientific knowledge informing recommendations for developers on how to limit nitrosamines in medicines.

## Introduction

The presence of *N*-nitroso-dimethylamine (NDMA) in valsartan from one active pharmaceutical ingredient (API) manufacturer was first reported in June 2018. Subsequently, an EU-wide review (so-called “referral procedure”) was initiated to assess the impact on the benefit-risk of valsartan-containing medicines. The referral was subsequently extended to cover all sartan medicines containing a tetrazole ring as part of their structure (1). In 2019, another EU review was initiated to review ranitidine-containing medicines in view of concerns over the presence of NDMA, degradation over time to form more NDMA and its potential endogenous formation in gastric-like conditions, which altogether led to the suspension of Marketing Authorisations of all ranitidine-containing medicinal products in the EU in 2020 (2).

In parallel, a general EU review [“Article 5(3)”] procedure (3) on presence of nitrosamines in human medicines was initiated to investigate the risk of nitrosamines in manufacturing of medicines and how these can be avoided. Subsequently, nitrosamines have been detected at low levels in products containing several other active substances including metformin and rifampicin (4).

## Why Were Nitrosamines Present in Medicines?

The EU reviews have identified a number of root causes leading to the presence of nitrosamines in medicines, which all have in common a reaction of a secondary or tertiary amine with a nitrosating agent leading to the formation of an N-nitrosamine. Frequently, the nitrosating agent was found to be sodium nitrite, often under acidic conditions during manufacture of the active pharmaceutical ingredient (API). In several sartan APIs, this occurred in the final step of the active substance manufacturing process during synthesis of the tetrazole ring leading to high levels of nitrosamines (1). However, there are also examples of nitrosamine formation during finished product manufacture, including during primary packaging. The use of contaminated reagents or solvents has also been associated with the presence of nitrosamines in medicines. Furthermore, degradation of the active substance (e.g., ranitidine) itself leading to nitrosamine impurities has been observed (5), and reactions of residual amine impurities with nitrosating sources in excipients or primary packaging are postulated to have contributed to NDMA contamination in metformin medicines (6). This summary of root causes is not exhaustive, but it illustrates the variety of reasons for presence of nitrosamines in medicines, and other root causes may still be discovered.

## Risk Assessment and Limits for Nitrosamines

At present, generally there are insufficient data on carcinogenic potency of nitrosamines in humans, although most substances were found to be carcinogenic *in vivo*. The potential risk for humans is therefore extrapolated from animal data and varies by orders of magnitudes for different nitrosamines.

As a precaution, conservative methods are employed to set limits for mutagenic carcinogens. Existing pharmaceutical guidance [ICH M7 (R1)] (7) covers mutagenic carcinogens and applies a threshold of toxicological concern (TTC) approach with defining a negligible risk level (a theoretical 10^−5^ excess lifetime risk of cancer) to set an acceptable intake (AI) for any mutagenic substance. The methods upon which the TTC is based are generally considered to be very conservative since they involve a simple linear extrapolation from the dose giving a 50% tumour incidence (TD50) to a 10^−5^ incidence, using TD50 data for the most sensitive species and most sensitive site of tumour induction. For most mutagenic chemicals, a generic TTC of 1.5 μg/day can be justified, however it excludes structural groups of such high potency, for which intakes even below the TTC of 1.5 μg/day potentially could be associated with a carcinogenic risk exceeding the 1 in 10^5^ incidences. Such chemicals are referred to as the “cohort of concern” and nitrosamines belong to this group. For nitrosamines, ICH M7(R1) recommends calculating a substance-specific AI using the TD50 or Benchmark Dose Limit (BMDL_10_) of animal carcinogenicity studies performed with the specific nitrosamine as “points of departure,” i.e., starting points for the calculations.

Linear extrapolation of cancer risk from animals to humans using Haber's law is a conservative approach agreed by human medicines committee (CHMP) of the EMA for setting limits for nitrosamines in pharmaceuticals based on life-time exposure to the impurity. However, ICH M7(R1) also allows upward adjustment of impurity levels in products intended for less than lifetime (LTL) use based on the linearity paradigm “dose × time = constant.” If this concept were applied to nitrosamines, this would result in an acceptance of much higher levels, especially in medicinal products used only short-term.

When looking at how to implement the recommendations provided in ICH M7(R1) in the case of nitrosamine impurities, the validity of the linearity paradigm was questioned by experts consulted as part of the Article 5(3) scientific review on nitrosamines of EMA, at least for highly mutagenic and carcinogenic nitrosamines. Therefore, CHMP recommended not making adjustments to limits for LTL use as a precautionary measure. This also took into account uncertainties about whether short-term intake of high levels of nitrosamines could acutely overwhelm the DNA repair capacities of the body. This would especially be the case for products used short term, which would allow much higher nitrosamine levels when using the LTL approach of ICH M7 (R1).

Therefore, the default approach for calculation of nitrosamine limits is based on the AI for lifetime exposure *as per* the principles of ICH M7(R1). However, there may be exceptional cases, such as critical medicinal products with limited therapeutic alternatives that would be at risk of shortage in case of recalls or disruption of supply if an AI for lifetime exposure is applied. In such cases, when a N-nitrosamine cannot be kept below the AI limit, higher limits may exceptionally be accepted by the relevant authorities, but only after having performed a benefit/risk evaluation, with the assessment being coordinated at EU level through the Scientific Committees of EMA to facilitate a harmonised approach across Member States.

When it comes to setting limits for new nitrosamines, should these be detected, the recommendations provide flexibility depending on the type of available data. The AI can be derived by extrapolation from available animal data. In the absence of adequate animal data, a class specific TTC for nitrosamines of 18 ng/day can be used, based on a worst-case scenario using TD_50_ data from all nitrosamines in the Lhasa Carcinogenicity Database, or structural activity relationship calculations can be performed using, e.g., carcinogenicity data from closely related structures. The AI limit will in any case require assessment by the CHMP, which will ensure harmonised limits based on a single AI for a given nitrosamine across all EU Member States.

In addition to the recommendations on setting limits, CHMP provided guidance on strategies to mitigate the presence of nitrosamines in human medicinal products. These include careful design of manufacturing processes to avoid nitrosamine formation, risk assessments for processes and raw materials, and implementation of appropriate control strategies. Further guidance was provided on calculating limits when multiple nitrosamines are present and on aspects of analytical methods for detection and quantification of nitrosamines at trace levels.

## Discussion

The EU regulatory network is working on implementing the recommendations from the Article 5(3) review for all chemical or biological human medicines authorised in the EU, including setting nitrosamine limits based on lifetime exposure, requesting MAHs to carry out risk assessments and develop control strategies, and testing medicinal products for which a risk has been identified.

Other initiatives include implementation of recommendations from a recently concluded lessons learnt exercise on sartans (8), which focus on additional scientific and regulatory guidance to better control nitrosamines and other impurities in the future. The outcomes of the Article 5(3) review together with the lessons learnt exercise strive to reduce the risk of nitrosamine contamination in all existing and new medicinal products in the EU.

As nitrosamine contamination affects patients worldwide, EU regulators, such as EMA, are working with international partners and will continue to take necessary measures to ensure that manufacturers and Marketing Authorisation Holders adhere to recommendations regarding nitrosamines and other impurities while ensuring that patients maintain access to safe and effective medicines.

## Data Availability Statement

The original contributions presented in the study are included in the article, further inquiries can be directed to the corresponding author/s.

## Author Contributions

RR and RF wrote the manuscript. RB, MF, TG, AS, MW, and RW participated in the final manuscript design and provided experts' opinion on the content and critical revision. All authors contributed to the article and approved the submitted version.

## Author Disclaimer

The views expressed in this article are the personal views of the author(s) and may not be understood or quoted as being made on behalf of or reflecting the position of the regulatory agency/agencies or organisations with which the author(s) is/are employed/affiliated.

## Conflict of Interest

The authors declare that the research was conducted in the absence of any commercial or financial relationships that could be construed as a potential conflict of interest.

## Publisher's Note

All claims expressed in this article are solely those of the authors and do not necessarily represent those of their affiliated organizations, or those of the publisher, the editors and the reviewers. Any product that may be evaluated in this article, or claim that may be made by its manufacturer, is not guaranteed or endorsed by the publisher.
